# The STING pathway does not contribute to behavioural or mitochondrial phenotypes in *Drosophila Pink1/parkin* or mtDNA mutator models

**DOI:** 10.1038/s41598-020-59647-3

**Published:** 2020-02-14

**Authors:** Juliette J. Lee, Simonetta Andreazza, Alexander J. Whitworth

**Affiliations:** MRC Mitochondrial Biology Unit, University of Cambridge, Cambridge Biomedical Campus, Hills Road, Cambridge, CB2 0XY United Kingdom

**Keywords:** Mechanisms of disease, Genetics of the nervous system

## Abstract

Mutations in *PINK1* and *Parkin/PRKN* cause the degeneration of dopaminergic neurons in familial forms of Parkinson’s disease but the precise pathogenic mechanisms are unknown. The PINK1/Parkin pathway has been described to play a central role in mitochondrial homeostasis by signalling the targeted destruction of damaged mitochondria, however, how disrupting this process leads to neuronal death was unclear until recently. An elegant study in mice revealed that the loss of *Pink1* or *Prkn* coupled with an additional mitochondrial stress resulted in the aberrant activation of the innate immune signalling, mediated via the cGAS/STING pathway, causing degeneration of dopaminergic neurons and motor impairment. Genetic knockout of *Sting* was sufficient to completely prevent neurodegeneration and accompanying motor deficits. To determine whether Sting plays a conserved role in *Pink1/parkin* related pathology, we tested for genetic interactions between *Sting* and *Pink1/parkin* in *Drosophila*. Surprisingly, we found that loss of *Sting*, or its downstream effector *Relish*, was insufficient to suppress the behavioural deficits or mitochondria disruption in the *Pink1/parkin* mutants. Thus, we conclude that phenotypes associated with loss of *Pink1/parkin* are not universally due to aberrant activation of the STING pathway.

## Introduction

Loss of function mutations in *PINK1* and *PRKN* cause familial parkinsonism, an incurable neurodegenerative disorder predominantly associated with the progressive loss of dopaminergic neurons in substantia nigra leading to loss of motor control. *PRKN* encodes a cytosolic ubiquitin E3 ligase, Parkin, and *PINK1* encodes a mitochondrially targeted kinase. Extensive evidence shows that they cooperate in signalling the targeted autophagic destruction of damaged mitochondria (mitophagy) as part of a homeostatic mitochondrial quality control process^1,2^.

Mitochondria are essential organelles that perform many critical metabolic functions but are also a major source of damaging reactive oxygen species and harbour pro-apoptotic factors. Hence, multiple homeostatic processes, such as mitophagy, operate to maintain mitochondrial integrity and prevent potentially catastrophic consequences. Such homeostatic mechanisms are particularly important for post-mitotic, energetically demanding tissues such as nerves and muscles.

The molecular details of PINK1/Parkin-induced mitophagy are well characterized in cultured cells, however, relatively little is known about mitophagy under physiological conditions *in vivo*^3–5^. Nevertheless, several studies provide evidence consistent with PINK1 and Parkin acting to remove mitochondrial damage *in vivo*. One study used a mass spectrometry-based analysis of mitochondrial protein turnover in *Drosophila*^6^, which revealed that fly PINK1 and Parkin selectively affect the degradation of certain mitochondrial proteins under physiological conditions. Another found that loss of *Prkn* in mice, which alone has very little phenotype^7,8^, exacerbated the phenotypic effects of a mitochondrial DNA mutator strain, provoking loss of dopaminergic neurons and motor deficits^9^.

Importantly, a subsequent study shed light on the mechanism by which loss of *Pink1/Prkn* leads to neurodegeneration in the presence of mtDNA mutations, or upon exposure to exhaustive exercise, as chronic or acute mitochondrial stresses, respectively^10^. This demonstrated that in the absence of *Pink1/Prkn* these mitochondrial stresses cause an aberrant inflammatory response mediated by the STING pathway, presumably via the release of mtDNA into the cytosol. Consequently, loss of *STING* completely prevented the inflammatory response and the resulting neurodegeneration and locomotor phenotypes^10^. These results strongly implicate the induction of STING-mediated inflammation in the pathogenic cause of Parkinson’s disease.

The recently identified *Drosophila Sting* ortholog has been shown to bind to cyclic-dinucleotides, in particular 2′3′-cGAMP, and trigger an immune response to bacterial and viral infection^11–14^, mediated by the IMD pathway and the transcription factor Relish (homologous to NF-κB). Consequently, *Drosophila* mutant for *Sting* showed a reduced survival upon infection. Interestingly, while aberrant activation of the IMD-Relish pathway has been shown to cause neurodegeneration and shortened lifespan in *Drosophila*^15^, transcriptional profiling has shown that innate immune signalling pathways are ectopically active in *Drosophila parkin* and *Pink1* mutants^16,17^.

The *Drosophila* models have been highly informative for interrogating the physiological role of PINK1/Parkin, primarily via genetic or pharmacological manipulations^17–21^ that can modify the robust neuromuscular phenotypes associated with loss of the *Pink1/parkin* orthologs^22–25^ (for review see^26^). Therefore, we sought to determine whether aberrant activation of the Sting-Relish immune signalling cascade may contribute to the neuromuscular degeneration phenotypes observed in *Drosophila Pink1/parkin* mutants. Surprisingly, we found that loss of *Sting* or *Relish* had no suppressing effect on the locomotor deficits or mitochondrial disruption in *Pink1* or *parkin* mutants. Moreover, *Sting* knockout did not affect the behavioural phenotypes associated with a fly mtDNA mutator model, nor the combined effect of mtDNA mutations in a *parkin* background. Hence, the central role of Sting in the induction of *Pink1/parkin* mutant phenotypes proposed for mammals is not conserved in *Drosophila*.

## Results

**Figure 1:**
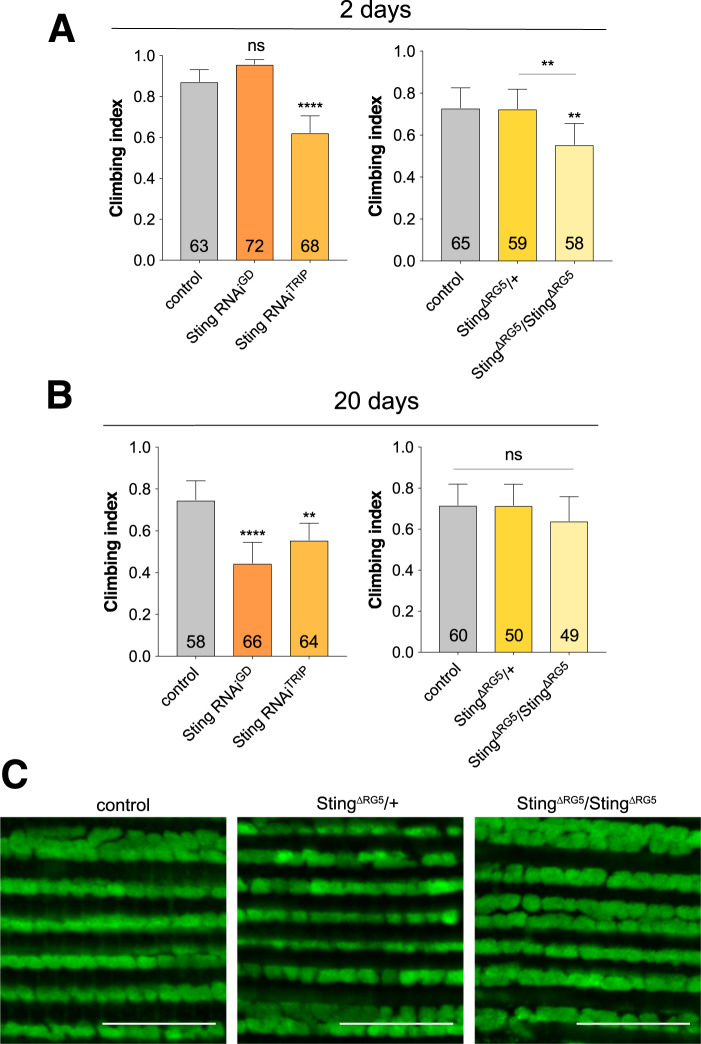
Loss of *Sting* has limited impact on neuromuscular phenotypes. Locomotor assays analysing climbing ability (negative geotaxis) in (**A**) young and (**B**) older adult flies of control and *Sting* knockdown (RNAi) or null (*Sting*^ΔRG5^) mutants. Charts show mean ± 95% confidence interval (CI); number of animals analysed is shown in each bar. Significance was measured by Kruskal-Wallis test with Dunn’s post hoc correction for multiple comparisons; **p < 0.01, ****p < 0.0001; ns, non-significant. Control genotype is *da-GAL4*/+. (**C**) Representative confocal microscopy analysis of mitochondria in flight muscles, immunostained with anti-ATP5A, in control (*w*^1118^) and *Sting* heterozygous and homozygous mutants. Scale bar = 10 µm.

*Drosophila Sting* mutants have recently been generated and, consistent with Sting’s role in triggering an innate immune response, shown to be more susceptible to infection^11–13^. As other organismal phenotypes were not reported, we first assessed whether loss of *Sting* may induce additional phenotypes associated with the neuromuscular system that might confound further genetic interaction analysis. To this end, we examined the motor behaviour and muscle integrity in *Sting* loss of function conditions. We assessed the impact of RNAi-induced loss of function using previously validated RNAi lines expressed via the ubiquitous driver *da-GAL4*. A small impact on climbing ability in young flies was observed with one RNAi transgene, which was also seen in homozygous *Sting* null (*Sting*^ΔRG5^) mutants (Fig. 1A). Aged *Sting*-RNAi flies showed a consistent, modest impact on climbing ability, but this was not evident in *Sting* mutants (Fig. 1B). Microscopy analysis of muscle and mitochondrial integrity did not reveal any obvious disruption in *Sting* mutants (Fig. 1C). Since loss of Sting did not appear to grossly affect neuromuscular integrity, we next assessed whether the activity of Sting contributed to the neuromuscular phenotypes in *Pink1/parkin* mutants.

**Figure 2:**
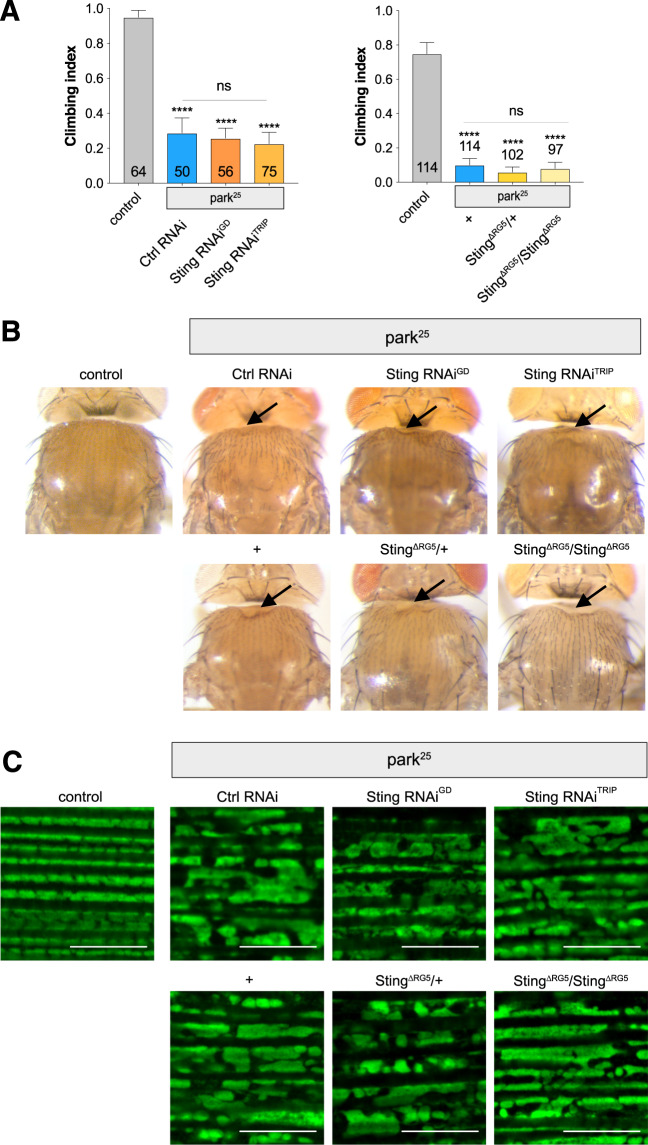
Loss of *Sting* does not modify *parkin* mutant phenotypes. (**A**) Analysis of locomotor (climbing) ability, (**B**) thoracic indentations, and (**C**) mitochondrial morphology in young *park*25 mutants combined with *Sting* knockdown or null mutations. Charts show mean ± 95% confidence interval (CI); number of animals analysed is shown in each bar. Statistical significance was measured by Kruskal-Wallis test with Dunn’s post hoc correction for multiple comparisons; ****p < 0.0001; ns, non-significant. Confocal microscopy images show flight muscle mitochondria immunostained with anti-ATP5A. Scale bar = 10 µm. Control genotypes are *da-GAL4*/+ for climbing, and *w*^1118^ for thoracic indentation and microscopy. Ctrl RNAi is *lacZ*-RNAi in the mutant background.

Combining all the manipulations of *Sting* (two RNAi transgenes, heterozygous and homozygous null mutations) with *parkin* null mutants (*park*^25^), we did not observe any modification (suppression or enhancement) of the *parkin* mutants climbing defect (Fig. 2A). Similarly, the thoracic indentations typically observed in *park*^25^ flies due to the degeneration of the underlying musculature, were still present in the absence of *Sting* (Fig. 2B). Consistent with this, we did not observe any improvement of the tissue or mitochondrial integrity in the flight muscles of *parkin* mutants by removal of *Sting* (Fig. 2C).

**Figure 3:**
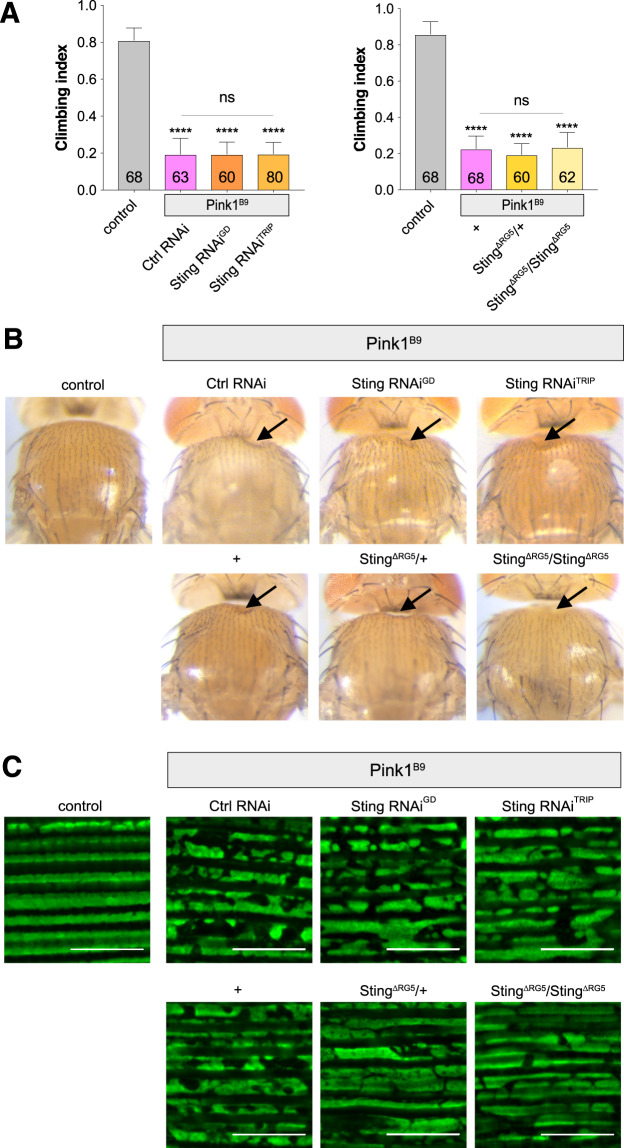
Loss of *Sting* does not modify *Pink1* mutant phenotypes. (**A**) Analysis of locomotor (climbing) ability, (**B**) thoracic indentations, and (**C**) mitochondrial morphology in young *Pink1*^B9^ mutants combined with *Sting* knockdown or null mutations. Charts show mean ± 95% confidence interval (CI); number of animals analysed is shown in each bar. Statistical significance was measured by Kruskal-Wallis test with Dunn’s post hoc correction for multiple comparisons; ****p < 0.0001; ns, non-significant. Confocal microscopy images show flight muscle mitochondria immunostained with anti-ATP5A. Scale bar = 10 µm. Control genotypes are *da-GAL4*/+ for climbing, and *w*^1118^ for thoracic indentations and microscopy. Ctrl RNAi is *lacZ*-RNAi in the mutant background.

We next assessed the contribution of Sting function towards *Pink1* mutant (*Pink1*^B9^) phenotypes. Similar to *parkin* mutants, loss of *Sting* failed to modify the climbing defect (Fig. 3A), thoracic indentations (Fig. 3B) or disruption of flight muscle and mitochondrial integrity (Fig. 3C) observed in *Pink1*^B9^ flies. Taken together, these results indicate that Sting does not contribute to the neuromuscular phenotypes observed in *Pink1/parkin* mutants.

**Figure 4:**
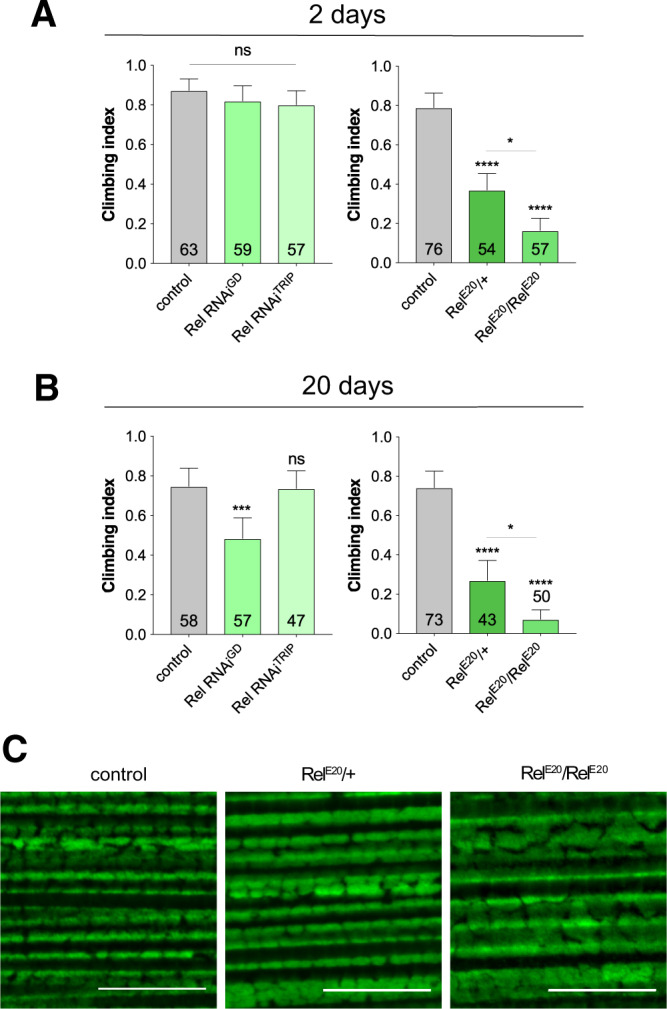
Loss of *Relish* causes mild locomotor deficits. Locomotor assays analysing climbing ability in (**A**) young and (**B**) older adult flies of control and RNAi knockdown or *Relish* mutant (*Rel*^E20^). Charts show mean ± 95% confidence interval (CI); number of animals analysed is shown in each bar. Statistical significance was measured by Kruskal-Wallis test with Dunn’s post hoc correction for multiple comparisons; *p < 0.05, ***p < 0.001, ****p < 0.0001; ns, non-significant. Control genotype is *da-GAL4*/+. (**C**) Representative confocal microscopy analysis of mitochondria in flight muscles, immunostained with anti-ATP5A, in control (*w*^1118^) and *Relish* heterozygous and homozygous mutants. Scale bar = 10 µm.

Considering that loss of *STING* in mouse completely abrogated the *Pink1/Prkn*-associated neurodegeneration and motor phenotypes provoked by additional mitochondrial stresses, we were surprised by the lack of suppression of *Pink1/parkin* phenotypes in flies. Therefore, to further interrogate the potential contribution of this pathway to *Pink1/parkin* pathology, we also analysed a downstream effector of the Sting-IMD pathway, the transcription factor Relish (Rel). While RNAi knockdown using two previously characterized transgenes^11,13^ elicited modest effect on climbing, *Rel* mutants (*Rel*^E20^) displayed a strong locomotor defect (Fig. 4A, B). However, analysis of flight muscles in these mutants did not reveal any major disruption of mitochondrial integrity (Fig. 4C).

**Figure 5:**
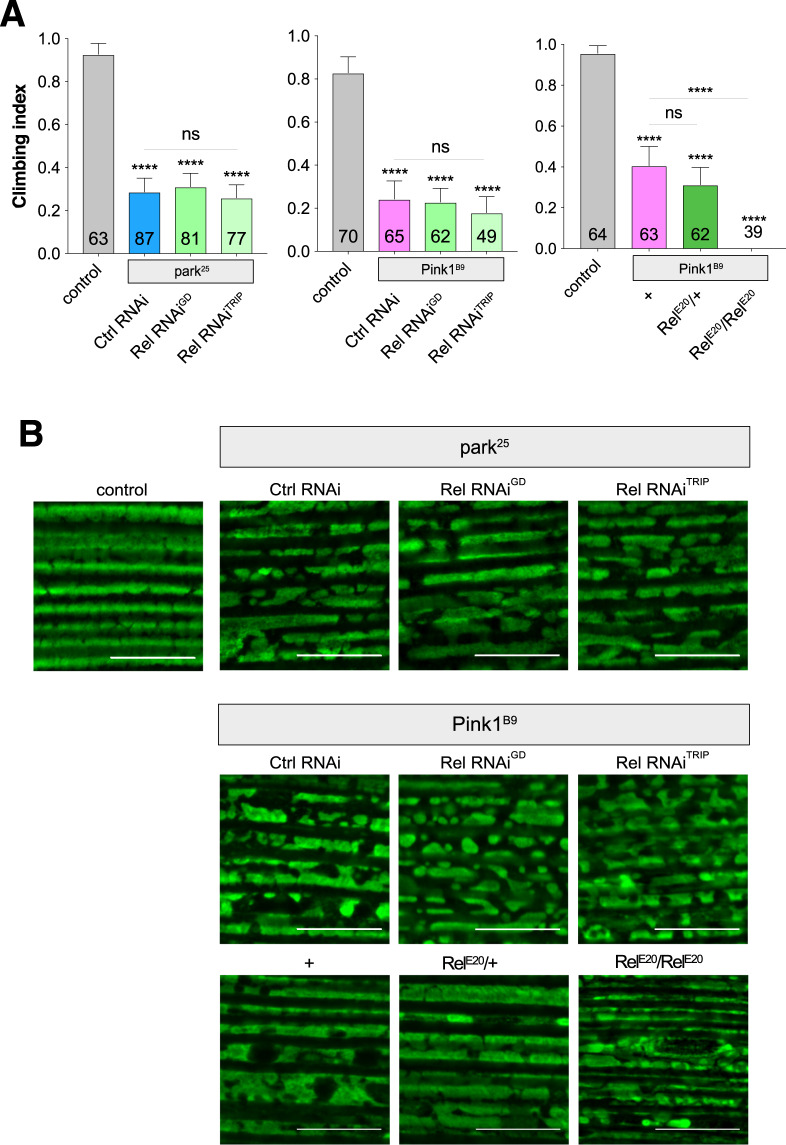
Loss of *Relish* does not rescue *Pink1* or *parkin* mutant phenotypes. (**A**) Analysis of locomotor (climbing) ability and (**B**) mitochondrial morphology in young *park*^25^ or *Pink1*^B9^ mutants combined with *Relish* knockdown or null mutations. Charts show mean ± 95% confidence interval (CI); number of animals analysed is shown in each bar. Statistical significance was measured by Kruskal-Wallis test with Dunn’s post hoc correction for multiple comparisons; ****p < 0.0001; ns, non-significant. Confocal microscopy images show flight muscle mitochondria immunostained with anti-ATP5A. Scale bar = 10 µm. Control genotypes are *da-GAL4*/+ for climbing, and *w*^1118^ for microscopy. Ctrl RNAi is *lacZ*-RNAi in the respective mutant background.

Similar to the *Sting* manipulations, RNAi knockdown of *Rel* did not modify the climbing deficit of *parkin* or *Pink1* mutants (Fig. 5A), nor did it noticeably affect the mitochondrial integrity in flight muscles (Fig. 5B). Indeed, in contrast to expectation, genetic loss of *Rel* enhanced the *Pink1* locomotor defect (Fig. 5A), although the mitochondrial integrity was not noticeably worsened in *Pink1*^B9^; *Rel*^E20^ flies (Fig. 5B).

**Figure 6:**
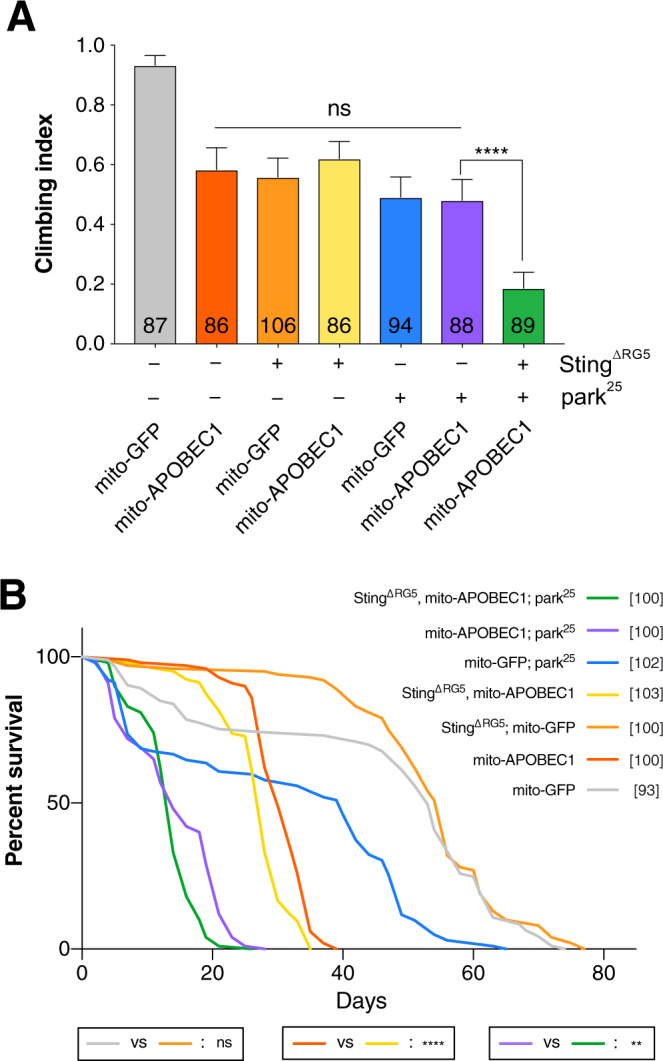
Loss of *Sting* does not ameliorate mtDNA *mutator*; *parkin* mutant combinations. Analysis of (**A**) locomotor (climbing) ability in young adults and (**B**) lifespan in flies combining *mito-APOBEC1* mtDNA mutator expression with or without *parkin* and/or *Sting*. Transgene expression was driven by *da-GAL4*. *Mito-GFP* expression was used as a control. Charts show mean ± 95% confidence interval (CI); number of animals analysed is shown in each bar for climbing, and beside the genotype key for lifespan. For climbing, statistical significance was determined by Kruskal-Wallis test with Dunn’s post hoc correction for multiple comparisons; and for lifespan, by Log-rank (Mantel-Cox) test; **p < 0.01, ****p < 0.0001; ns, non-significant.

In a final effort to assess whether the *Drosophila* Pink1/parkin-Sting axis acts in an analogous fashion to mice, we sought to recapitulate the conditions assessed by Sliter *et al*.^10^ and test the role of Sting when an additional mitochondrial stress is combined with *parkin* loss-of-function. To do this, we used our previously established mtDNA mutator model (mito-APOBEC1), which generates high levels of deleterious mtDNA mutations in somatic tissues, disrupting mitochondrial function and causing motor defects and shortened lifespan^27^. Notably, the loss of *parkin* or *Sting* did not exacerbate the impact of mito-APOBEC1 alone on locomotor function (Fig. 6A). Furthermore, the combination of the mtDNA mutator in a *parkin*; *Sting* double mutant background, in stark contrast to the results in mice^10^, enhanced the climbing deficit (Fig. 6A). Similarly, while loss of *Sting* alone did not affect normal lifespan, it significantly enhanced the shortened lifespan of the mito-APOBEC1 model or the combination of mito-APOBEC1 with *parkin* loss-of-function (Fig. 6B), consistent with the locomotor analysis.

Thus, together the above data suggest that the Sting pathway, although proposed to be mediating motor and neurodegenerative defects in *Prkn*^−/−^ mice, do not similarly contribute to the neuromuscular defects observed in *Pink1/parkin* mutant flies.

## Discussion

Understanding the pathogenic mechanisms by which loss of function mutations in PINK1 and Parkin lead to neurodegeneration in Parkinson’s disease is central to defining better disease-modifying therapies. While tremendous advances have been made in uncovering the molecular mechanisms of PINK1/Parkin function *in vitro* and in cell culture models, understanding the consequences of this dysfunction on neuronal demise must be studied *in vivo*, in the complex milieu of organismal biology. This has been severely hampered by the lack of robust phenotypes in *Pink1/Prkn* knockout mice. In contrast, *Drosophila* models have provided substantial insights in this realm as fly *Pink1/parkin* mutants exhibit extensive disruption of the neuromuscular system presenting, amongst other phenotypes, profound deficits in locomotor behaviours, apoptotic degeneration of flight muscles, progressive degeneration of dopaminergic neurons, all accompanied by morphological and functional breakdown of mitochondria. Consequently, genetic studies using the fly models, primarily using suppression or enhancement of the mutant phenotypes as a sensitive readout, have elucidated several important and conserved features of PINK1/Parkin biology^17–21,26^.

Recent studies have shed new light on the *in vivo* role of PINK1/Parkin in vertebrates, and the context in which loss of *Pink1/Prkn* can reveal pathogenic phenotypes. First, combining *Prkn* knockout mutants with a mtDNA mutator strain selectively led to degeneration of nigral dopaminergic neurons, decline in motor ability and increased mitochondrial dysfunction^9^. Extending these observations, Sliter *et al*.^10^ revealed that this *Prkn*^−/−^; *mutator* combination (or *Pink1*^−/−^; *mutator*) provoked an aberrant innate immune response mediated by the STING pathway, suggesting that the systemic inflammatory response ultimately caused the dopaminergic neurodegeneration and motor deficits. Indeed, genetic loss of *STING* was sufficient to completely prevent the inflammation, motor defect and neurodegeneration in the *Prkn*^−/−^; *mutator* mice. These findings established the STING pathway and, more broadly, aberrant innate immune signalling, as a pathogenic cause and a highly attractive therapeutic target. Moreover, additional work has also implicated *Pink1/Prkn* mutations in inducing aberrant inflammation, albeit via adaptive immunity^28^. However, while the PINK1/Parkin pathway is clearly an ancient mechanism regulating mitochondrial quality control, our data indicate that Sting does not appear to be a fundamental, conserved feature of PINK1/Parkin biology.

The question arises why loss of *Sting* does not suppress *Pink1/parkin* phenotypes in flies when it is capable of completely preventing pathology in mice? At this stage, the answer is unknown and rather puzzling given that innate immune signalling is dysregulated in *Pink1/parkin* mutants^16,17^, and Sting performs an analogous function in flies as it does in vertebrates^11^. One possibility is that the aberrant innate immune activation observed in *parkin* and *Pink1* mutant flies is not mediated by the presence of cytosolic DNA or activation of the Sting pathway. Moreover, investigating whether induction of mtDNA mutations is required to trigger the innate immune response, as indicated by Sliter *et al*., our data show that even in the presence of a mtDNA mutator, the Sting immune cascade did not contribute to the neuromuscular phenotypes caused by loss of *Pink1/parkin* in flies. An alternative interpretation is that the *Pink1/parkin* phenotypes are not due to aberrant immune signalling and this may be an epiphenomenon. Supporting this view, many studies have established that loss of Pink1/parkin in flies causes catastrophic mitochondrial disruptions, triggering cell-autonomous apoptosis^22–24^.

Considering this, it isn’t clear from current data why either exhaustive exercise or increased mtDNA mutations should trigger an innate immune response that is mitigated by PINK1/Parkin in mice. In the mouse model, the involvement of STING implicates the presence of cytosolic DNA as a trigger. The evidence from Sliter *et al*. suggests that exhaustive exercise or mtDNA mutations is sufficient to induce mitophagy, which if not properly executed by Pink1/Parkin leads to the release of mtDNA and activation of STING signalling. However, it remains unclear how these mitochondrial stresses in the absence of *Pink1/Prkn* lead to release of mtDNA – presumably by loss of integrity and rupture of the mitochondrial boundary membranes. The observed increase in mitophagy in mouse cardiac muscle upon exhaustive exercise is again intriguing as this tissue shares striking structural and functional homology with *Drosophila* flight muscles, further increasing the puzzle as to why the role of Sting does not appear to be a conserved feature of Pink1/parkin biology in flies. Clearly, further work is necessary in order to fully understand the mechanisms linking mitochondrial disruption and immune activation across species.

## Methods

### *Drosophila* stocks and husbandry

Flies were raised under standard conditions in a humidified, temperature-controlled incubator with a 12 h:12 h light:dark cycle at 25 °C, on food consisting of agar, cornmeal, molasses, propionic acid and yeast. Transgene expression was induced using the ubiquitous *da-GAL4* driver. The following strains were obtained from the Bloomington *Drosophila* Stock Center (RRID:SCR_006457): *w*^1118^ (RRID:BDSC_6326), *da-GAL4* (RRID:BDSC_55850), *Sting*^TRiP^ (RRID:BDSC_31565), *Relish*^TRiP^ (RRID:BDSC_33661), *Relish*^E20^ (RRID:BDSC_9457), *UAS-mito-HA-GFP* (RRID:BDSC_8443); and the Vienna Drosophila Resource Center (RRID:SCR_013805): *Sting*^GD^ (P{GD1905}v4031), *Relish*^GD^ (P{GD1199}v49413), and *lacZ-*RNAi (P{GD936}v51446) used as a control RNAi. Other lines were kindly provided as follows: *Sting*^ΔRG5^ from A. Goodman^11^, *Pink1*^B9^ mutants from J. Chung^24^, and the *park*25 mutants and *UAS-mito-APOBEC1* have been described previously^23,27^. All experiments were conducted using male flies.

### Locomotor and lifespan assays

The startle induced negative geotaxis (climbing) assay was performed using a counter-current apparatus. Briefly, 20–23 males were placed into the first chamber, tapped to the bottom, and given 10 s to climb a 10 cm distance. This procedure was repeated five times (five chambers), and the number of flies that has remained into each chamber counted. The weighted performance of several group of flies for each genotype was normalized to the maximum possible score and expressed as *Climbing index*^23^.

For lifespan experiments, flies were grown under identical conditions at low-density. Progeny were collected under very light anaesthesia and kept in tubes of approximately 20 males each, around 100 in total. Flies were transferred every 2–3 days to fresh medium and the number of dead flies recorded. Percent survival was calculated at the end of the experiment after correcting for any accidental loss.

### Immunohistochemistry and sample preparation

For immunostaining, adult flight muscles were dissected in PBS and fixed in 4% formaldehyde for 30 min, permeabilized in 0.3% Triton X-100 for 30 min, and blocked with 0.3% Triton X-100 plus 1% bovine serum albumin in PBS for 1 h at RT. Tissues were incubated with ATP5A antibody (Abcam Cat# ab14748, RRID:AB_301447; 1:500), diluted in 0.3% Triton X-100 plus 1% bovine serum albumin in PBS overnight at 4 °C, then rinsed 3 times 10 min with 0.3% Triton X-100 in PBS, and incubated with the appropriate fluorescent secondary antibodies overnight at 4 °C. The tissues were washed 2 times in PBS and mounted on slides using Prolong Diamond Antifade mounting medium (Thermo Fischer Scientific).

### Microscopy

Fluorescence imaging was conducted using a Zeiss LSM 880 confocal microscope (Carl Zeiss MicroImaging) equipped with Nikon Plan-Apochromat 100×/1.4 NA oil immersion objectives. Images were prepared using Fiji software (Fiji, RRID:SCR_002285). For thoracic indentations, images were acquired using a Leica DFC490 camera mounted on a Leica MZ6 stereomicroscope.

### Statistical analysis

For behavioural analyses, Kruskal-Wallis non-parametric test with Dunn’s post-hoc correction for multiple comparisons was used. For statistical analyses of lifespan experiment a Log-rank (Mantel-Cox) test was used. Analyses were performed using GraphPad Prism 8 software (RRID:SCR_002798).

## Data Availability

All data that support the findings of this study are available on reasonable request to the corresponding author. The contributing authors declare that all relevant data are included in the paper.
